# Empowering Physical Functions in Older Women With Sarcopenia Through Aomori Gymnastics: A Prospective, Observational, Nested Case-Control Study in Aomori Prefecture

**DOI:** 10.7759/cureus.79988

**Published:** 2025-03-03

**Authors:** Kentaro Yoshida, Toyohiro Hamaguchi, Kazuaki Masuda, Eiichi Tsuda, Mikio Hiura, Masahiro Abo

**Affiliations:** 1 Department of Rehabilitation Medicine, Jikei University School of Medicine, Tokyo, JPN; 2 Center for Brain and Health Sciences, Aomori University, Aomori City, JPN; 3 Department of Rehabilitation, Graduate School of Health Sciences, Saitama Prefectural University, Saitama, JPN; 4 Department of Rehabilitation Medicine, Hirosaki University Graduate School of Medicine, Hirosaki City, JPN; 5 Department of Neurosurgery, Aomori Shintoshi Hospital, Aomori City, JPN

**Keywords:** aomori gymnastics, frailty, older adults, physical activity, sarcopenia

## Abstract

Background: Japan faces rapid population aging, resulting in significant health challenges such as sarcopenia and frailty, which affect the independence and quality of life of older adults. This study evaluated the effectiveness of Aomori gymnastics, a culturally tailored exercise program, in improving physical function in older women with sarcopenia, over a three-month period.

Methods: This prospective observational study included 24 older women divided into three groups based on their living conditions: community-dwelling participants attending preventive care programs (Groups 1 and 2) and residents of a long-term care facility (Group 3). The participants performed Aomori gymnastics daily for three months. Physical function was assessed at baseline, 1 month, and three months using the Short Physical Performance Battery (SPPB), grip strength, curl-ups, forward bend from a long sitting position, one-leg stance, and 10-m walk tests. Data were analyzed using generalized linear mixed models and the Mann-Whitney U test.

Results: Significant improvements in SPPB scores were observed between the sarcopenia and non-sarcopenia groups at baseline and one month (p < .05), these differences were not observed at three months. Participants with sarcopenia showed marked improvement in physical function, approaching the level of those without sarcopenia after three months of daily exercise. Adherence was higher among long-term care facility residents than among community-dwelling participants, which is likely due to the severe winter weather in the Aomori Prefecture.

Conclusion: Aomori gymnastics is an effective and low-cost intervention that can significantly improve physical function in older women with sarcopenia. Implementing this program in communities and care facilities can enhance the quality of life and independence of older adults, particularly in regions with harsh weather conditions.

## Introduction

Japan is currently facing an unprecedented rate of population aging, representing one of the most rapidly aging societies worldwide [1]. This societal shift has given rise to numerous medical and socioeconomic challenges, particularly age-related health concerns of sarcopenia and frailty. Sarcopenia and frailty significantly impact the independence and quality of life of older adults, predisposing them to disabilities [2]. Sarcopenia not only affects the individual but also represents a major societal issue, contributing to increased healthcare costs and caregiver shortages [3,4].

Sarcopenia, characterized by age-related loss of muscle mass and strength [4,5], directly contributes to functional limitations, such as difficulties in performing daily tasks like rising from a chair, walking, or lifting objects. These functional impairments pose significant barriers to maintaining independence and often lead to a decline in activities of daily living (ADLs) [5,6]. Frailty, a condition marked by physical vulnerability, may also involve cognitive decline and psychological instability, contributing to functional limitations [7]. The effects of sarcopenia and frailty are prominently manifested in activity restrictions [4,8]. The functional limitations predispose older adults to reduced opportunities to engage in hobbies or social activities. This activity restriction affects physical and mental well-being, raises concerns about social isolation, and increases depressive symptoms.

Sarcopenia and frailty heighten the risk of falls, fractures, and other health problems among older adults, ultimately leading to increased healthcare costs [9,10]. The rapidly progressing demographic shift towards an aging population, coupled with a declining workforce, makes it increasingly challenging to provide the necessary resources for healthcare and long-term care. In Japan, caregiver shortages are a significant issue, and the mounting burden on families has socioeconomic implications and threatens the mental and physical well-being of caregivers. Consequently, sarcopenia and frailty are critical challenges that cannot be ignored in Japan's aging population. A comprehensive understanding of these issues from a social medicine perspective and the implementation of effective interventions are essential to enabling older adults to lead healthier and more independent lives [11].

While increasing physical activity has been shown to prevent sarcopenia [12], in certain areas of Japan, characterized by population decline and heavy snowfall, older adults may lack access to adequate physical therapy programs, with adherence to exercise programs posing a significant challenge owing to factors such as lack of motivation, social isolation, and cultural barriers. Innovative and engaging exercise interventions that incorporate culturally relevant elements may enhance adherence to exercise therapy and promote long-term adoption of an active lifestyle. In this context, the present study investigated the effects of the 'Aomori gymnastics,' a culturally tailored exercise routine developed in the Aomori region of Japan. Previous intervention studies for sarcopenia have primarily focused on resistance training protocols, protein supplementation, or multicomponent exercise programs delivered in clinical settings. While effective, these conventional approaches often face challenges in long-term adherence, particularly in rural areas with severe weather conditions. In contrast, Aomori gymnastics is performed on a specially composed piece of music created by a local musician, and the lyrics highlight the geographical features and local specialties of Aomori. By integrating traditional elements from the famous "Nebuta" festival, this program aims to promote physical activity while fostering a sense of cultural identity and enjoyment among older adults, potentially enhancing compliance compared to standard exercise protocols. Unlike typical resistance training programs requiring specialized equipment or supervision, Aomori gymnastics can be performed independently at home with minimal space requirements, making it particularly suitable for regions with limited access to fitness facilities or rehabilitation services.

The central hypothesis of this study was that a culturally tailored, low-intensity exercise program (Aomori gymnastics) specifically designed for the elderly population would significantly improve physical function in older adults with sarcopenia, potentially bridging the gap between those with and without sarcopenia. We hypothesized that the cultural relevance and accessibility of Aomori gymnastics would enhance exercise adherence, leading to improved functional outcomes, even in regions with challenging weather conditions that typically limit physical activity among the elderly. Furthermore, we predicted that these improvements would be observable within a relatively short timeframe (three months) and would be particularly pronounced among individuals with sarcopenia at baseline.

## Materials and methods

Study overview

**Figure 1:**
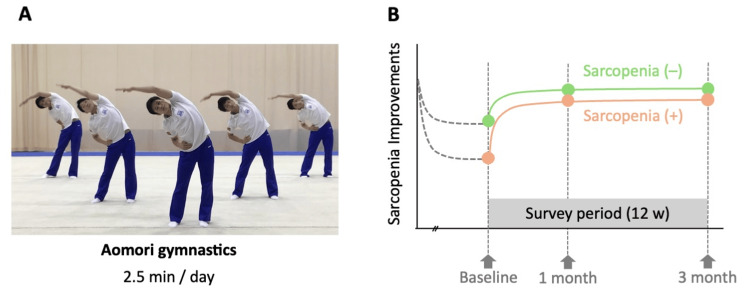
The conceptual diagram and protocol of this study The conceptual diagram and protocol of this study were designed to examine the differences in the effects of Aomori gymnastics on the physical condition of participants with or without sarcopenia. A: A still picture from the video demonstrating Aomori Gymnastics. The participants watched this video and performed the exercise of fully raising one leg in a standing position. B: All participants performed daily Aomori gymnastics at home or in a care facility for 2.5 minutes each time, continued for three months. Participants were classified according to whether they had sarcopenia, and the effects of Aomori exercises were compared in the analysis.

This prospective, observational study that investigated the effects of the "Aomori gymnastics" routine, which was conducted as part of a care prevention program in the Aomori region (Figure 1). Assessments were performed by the research staff at three time points: baseline (prior to starting the exercise program), 1 month, and 3 months. This study aimed to evaluate changes in physical function over a three-month period of participation in the Aomori gymnastics program.

Study participants

This study recruited older adults who participated in a physical activity program across three facilities between January 2023 and September 2023. The eligibility criteria were as follows: (1) age ≥ 65 years, (2) not requiring constant care for activities of daily living, and (3) ability to perform exercise routines independently. The exclusion criteria were (1) musculoskeletal conditions that precluded exercise participation and (2) severe cognitive impairment. As this was not a clinical study conducted in a medical setting, the medical parameters were not considered. 

Potential participants were screened for eligibility, and those who met the criteria were enrolled after they provided written informed consent. Statistical sample size calculations were not performed. However, we conducted post-hoc power and effect size analyses using the results of the 24 patients in this study using G*Power version 3.1 (https://www.gpower.hhu.de). We calculated post-hoc power critical z = 1.64, 1-β = .84, and effect size h = .20 from the z-test.

The three facilities were categorized into Groups 1−3, each representing distinct geographic and institutional characteristics within Aomori Prefecture. Group 1 included community-dwelling older adults attending a preventive care program (community salons, "Kayoi-no-ba") held at a public community center in central Aomori City between May and July 2023. Group 2 also comprised community-dwelling older adults attending a similar preventive care program at a community center between January and March 2023, a period characterized by heavy snowfall. Group 3 included older adults residing in a long-term care facility situated in western Aomori Prefecture, and data were collected between July and September 2023. The facility types also differed significantly in structure: the community centers (Groups 1 and 2) were public spaces with limited exercise equipment and variable attendance policies, while the long-term care facility (Group 3) featured dedicated rehabilitation areas, on-site healthcare staff, and structured daily activity schedules. For Groups 1 and 2, the "Kayoi-no-ba" were community-based programs aimed at promoting health and preventing care needs among independently living older adults who needed to travel to the centers regardless of weather conditions. Group 3 participants were recruited from a long-term care facility with 24-hour staff support, which allowed for the investigation of potential differences between community and institutionalized settings in terms of program implementation, adherence, and outcomes.

Aomori gymnastics

"Aomori gymnastics" is a 2.5-minute exercise routine designed to effectively stimulate muscles and joints in the entire body. It comprised 13 movements demonstrated by students from the gymnastics club at Aomori University and shown to the participants in video form. The movements were (1) shoulder horizontal abduction, (2) shoulder flexion/extension, (3) elbow flexion/extension, (4) back workouts, (5) trunk lateral flexion, (6) trunk rotation, (7) standing forward bend, (8) chest expansion exercises, (9) hip abduction, (10) hip flexion, (11) knee flexion, (12) ankle plantar flexion, and (13) marching in place. To promote adherence to the routine and prevent boredom, the following nine challenges were incorporated into the routine: (1) dual-task challenge, (2) attention-demanding tasks, (3) playful movements, (4) stepping pattern control, (5) bilateral jumping, (6) unilateral jumping, (7) "Nebuta" movements, (8) single-leg stance balance, and (9) deep breathing. The integration of "Nebuta," one of Japan's most renowned summer festivals with a long-standing tradition in Aomori, adds to the cultural relevance and engagement of the exercise program.

Outcomes

The physical function of each participant was assessed before the exercise intervention and at one and three months after the survey period. The assessment measures were based on the "New Physical Fitness Test" [13], which is a standardized test battery used in Japan. The measures included (1) grip strength, (2) curl-ups (trunk muscle strength/endurance), (3) forward bend from a long sitting position (trunk flexibility), (4) one-leg stance with eyes closed (static balance), (5) 10-m walk (gait speed test), and (6) the Short Physical Performance Battery (SPPB). SPPB is a composite measure that evaluates lower extremity function through tests of standing balance, gait speed, and repeated chair stands [14,15]. The balance tests evaluate the ability of an individual to maintain standing balance in three progressively more challenging positions: side-by-side stand, semi-tandem stand, and tandem stand. Participants attempted each stance, and the time they could hold the position was scored according to predetermined criteria. For the gait speed test, participants were instructed to walk at their usual pace over a 4 m course twice. The gait speed score was calculated from the average time taken to complete the two trials. The chair stand test assessed lower body strength as participants were asked to rise from a seated position to a full standing position five times consecutively, as quickly as possible. The time taken to complete the five stands was scored according to age- and sex-specific norms (Table 1). All assessments were conducted by a licensed physical therapist who was not involved in the study, ensuring an unbiased evaluation of the participants' performance. Standard instructions and protocols were followed for each test component.

**Table 1:** Scoring criteria for SPPB The total Short Physical Performance Battery  (SPPB) score ranges from 0 (worst) to 12 (best) as a summation of the category scores.

Subtest	Time (sec)	Points
Balance Test		
Side-by-Side Stand	>10	1
Semi-Tandem Stand	>10	1
Tandem Stand	>10	2
	3–10	1
	unable	0
Gait Speed Test	< 4.82	4
	4.82– 6.20	3
	6.21–8.7	2
	>8.7	1
	unable	0
Repeated Chair Stands Test	≦ 11.19	4
	11.20–13.69	3
	13.70–16.69	2
	>16.70	1
	≧60 or unable	0

Statistical analysis

To estimate the effectiveness of Aomori gymnastics, participants were classified according to their sarcopenia status using the criteria of the Asian Working Group for Sarcopenia at baseline, SPPB score <9, and five times sit-to-stand test (FTSST) >12 sec [16]. This classification allowed us to assess the intervention's impact more precisely within homogeneous subgroups defined by their baseline clinical history. We employed a generalized linear mixed model (GLMM) to analyze the impact of the presence or absence of sarcopenia and intervention periods on the physical variables [17]. To assess the adequacy and fit of our model, we used both the Akaike Information Criterion (AIC) and the Bayesian Information Criterion (BIC). The Mann-Whitney U test was performed for each variable and each time period combination to compare the differences between the sarcopenia and non-sarcopenia groups. 

Data analysis was conducted using Python (version 3.8; Wilmington, DE, USA). We used the ‘pandas library’ (version 1.2.0) for data preprocessing and the ‘statsmodels’ library (version 0.12.0) to construct GLMMs using the ‘mixedlm’ function. The flexibility of Python allows for rigorous data manipulation and detailed modeling. 

Ethical considerations

All participants were assured that the collected data would be anonymized and that their privacy would be protected. All participants provided written informed consent. All researchers signed a consent form to ensure the protection of participants’ privacy. The study protocol was approved by the Research Ethics Committee of Aomori Shintoshi Hospital (R04-003).

## Results

Baseline characteristics

**Figure 2:**
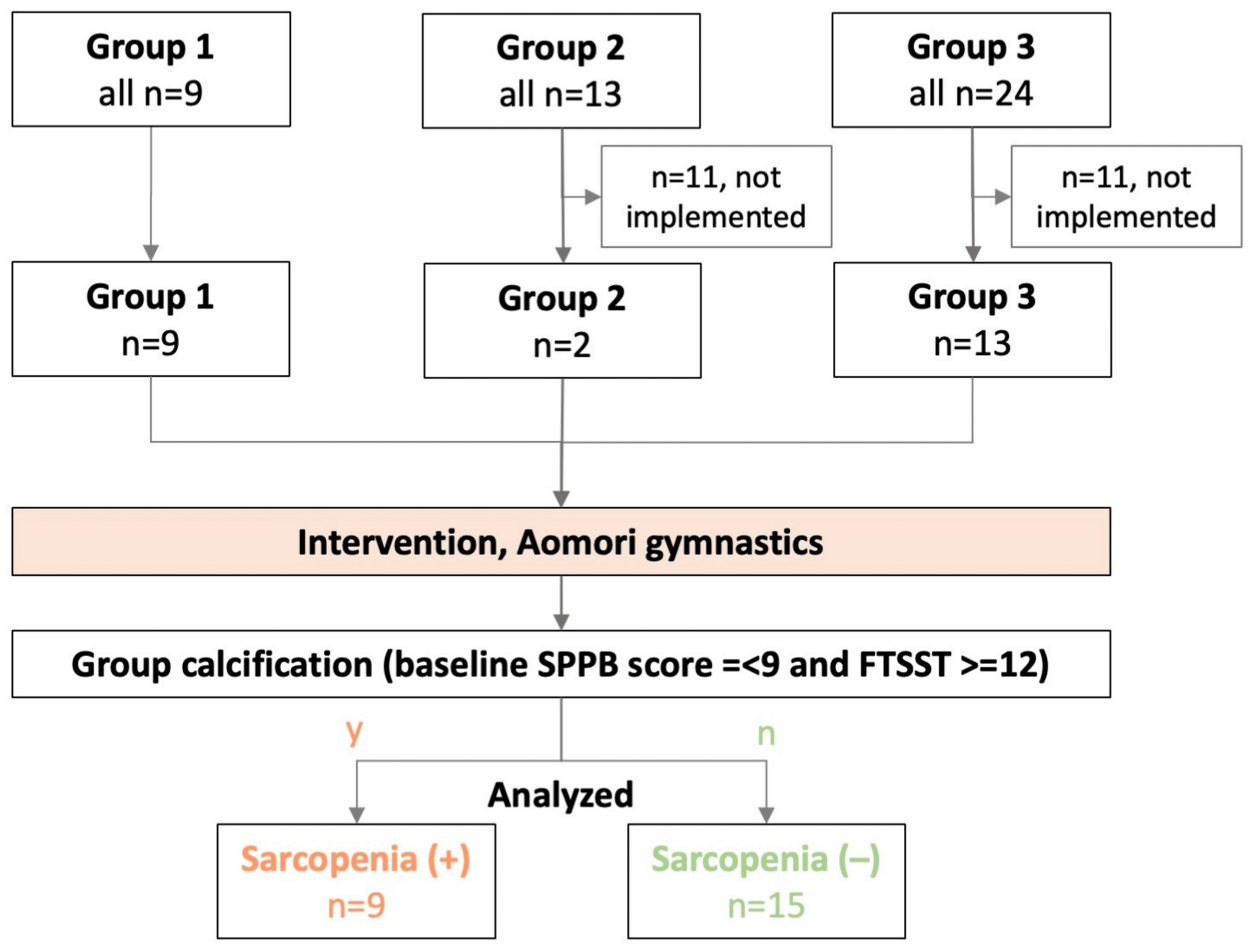
Flowchart of the processing protocol for the inclusion and classification of participants applied in this study Two community-dwelling elderly individuals and one geriatric care facility resident participated in this study. All participants performed the Aomori exercises. Participants were also divided into groups for data analysis according to the Short Physical Performance Battery (SPPB) score and self-adapted walking speed, with or without sarcopenia.

The baseline characteristics and performance metrics of the participants, grouped by their intervention, are summarized in Table 2. Twenty-six older women participated in the study: Group 1 (n=9), Group 2 (n=2), and Group 3 (n=13) (Figure 2). Participants in Groups 1 and 2 were elderly women living independently at home and fully capable of performing ADLs without assistance. In contrast, participants in Group 3 were elderly women residing in a care facility who required assistance with their ADL. The overall median age was 82.5 years (interquartile range (IQR) 78.0-86.2).

**Table 2:** Baseline summary of measures for participants by Group The data are from the survey conducted prior to the start of Aomori Gymnastics, and the values represent the median and the first and third quartiles. To comparing observed and expected frequencies among presence and absence of sarcopenia, Chi-squared test and Mann-Whitney U test. *p<.05. The effect size is reported as r. FTSST; Five-times-sit-to-stand test, SPPB; Short Physical Performance Battery, SLST; Single Leg Stance Test.

Measurements	Group 1	Group 2	Group 3	Sarcopenia (+)	Sarcopenia (-)	Total	Statistics	p	effect size
Participants (n)	9	2	13	9	15	24	χ2=0	1	df=1
Age (yrs)	82 (79, 83)	73 (71, 74)	86 (82, 88)	81 (77, 85)	83 (80, 87)	83 (78, 86)	U=43	.55	0.41
Total exercise over time	0 (0, 0)	81 (74, 87)	90 (42, 95)	93 (26, 27)	0 (0, 56)	20 (0, 93)	U=14	.04*	0.19
Sarcopenia (+ : –)	0 : 9	0 : 2	9 : 4	-	-	9 : 15	-	-	-
Grip Power (kg)	20.6 (20.1, 22.9)	21.9 (21.2, 22.6)	17.4 (14.9, 21.0)	16.2 (14.5, 21.0)	20.1 (17.3, 23.8)	20.2 (17.2, 22.7)	U=41	.14	r=0.75
FTSST (sec)	7.8 (7.3, 7.9)	8.3 (6.9, 9.7)	13.6 (12.6, 14.6)	14.3(13.1, 16.3)	7.7 (7.45, 9.1)	9.3 (7.6, 13.4)	U=4	.01*	r=0.07
Sit-up Test (times)	6.0 (.0, 9.0)	8.5 (8.2, 8.8)	.0 (.0, .0)	0 (0, 1)	2 (0, 6)	.0 (.0, 6.5)	U=71	.17	r=0.67
SPPB	12.0 (12.0, 12.0)	12.0 (12.0, 12.0)	8.0 (7.5, 10.0)	9 (9, 10)	12 (11, 12)	11.0 (8.2, 12.0)	U=51	.01*	r=0.93
SRT (cm)	39.0 (35.5, 40.0)	38.0 (37.5, 38.5)	27.0 (22.2, 33.8)	25.0 (17.0, 30.8)	35 (30, 38)	36.0 (28.0, 39.0)	U=37	.07	r=0.83
SLST (sec)	3.9 (2.8, 5.5)	13.6 (8.3, 18.8)	1.0 (.6, 1.8)	1.6 (1.0, 2.1)	2.3 (1.9, 4.45)	2.4 (1.0, 3.7)	U=42	.11	r=0.76
10-m Walk Test (sec)	5.8 (5.4, 6.0)	5.6 (5.5, 5.6)	10.0 (8.3, 13.4)	9.3 (8.9, 11.2)	5.6 (5.2, 6.5)	6.4 (5.7, 9.8)	U=4	.01*	r=0.06

All participants across all groups were prescribed and instructed to perform the Aomori gymnastics exercises daily. However, adherence varied significantly between groups. In terms of total exercises performed over time, the median total exercises across all groups was 20.0 times (IQR .0-93.0). None of the women in Groups 1 and 2 had sarcopenia; nine women in Group 3 had sarcopenia. All participants with sarcopenia (+) (n=9; 86 (82-88) years old) exercised 87 (57-94) times, whereas those without sarcopenia (-) (n=15; 81 (77-85) years old) showed lower adherence with a median of 0 (0-56) times. This stark difference in adherence is primarily attributed to the fact that most participants without sarcopenia were community-dwelling (Groups 1 and 2) and faced significant barriers to regular exercise participation due to the severe winter conditions in Aomori Prefecture. 

Efficacy outcomes

**Figure 3:**
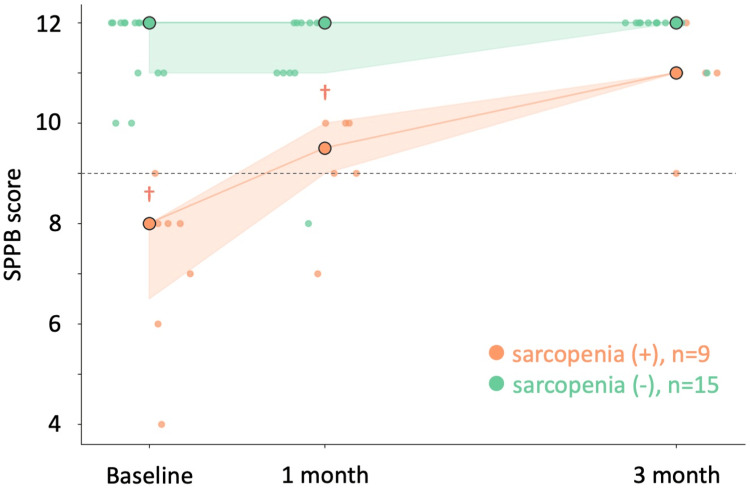
Scatter plots of the SPPB score classified by the presence or absence of sarcopenia during the survey of Aomori gymnastics intervention Plots of changes in the scores compared with the survey periods of all patients. Orange dots indicate the Short Physical Performance Battery (SPPB) scores of participants with sarcopenia, and green dots indicate those of participants without sarcopenia. Large dots are the median in each period. The error areas represent the first and third quartiles. ^†^p < 0.05, difference between the groups (with or without sarcopenia) at the same period by Mann-Whitney U test performed on a generalized linear mixed model.

The impact of sarcopenia on physical performance metrics was analyzed using GLMMs, with survey period as a temporal factor and sarcopenia as a fixed factor (Tables 3, 4). Significant interactions between period and sarcopenia were observed in the SPPB score (β = .93, SE = .43, p < .05, AIC = 299.79, BIC = 306.29, log-likelihood = -146.89) (Figure 3). There was no interaction between time period and participant group in the other physical performance metrics. The sarcopenia group had significantly lower performance in the sit-up test (β = -2.74, SE = 1.06, p < 0.05), lower SPPB scores (β = 3.68, SE = .97, p < .001), less grip power (β = 3.94, SE = 1.38, p < .01), lower FTSST scores (β = -6.95, SE = 0.98, p < 0.001), lower sit and reach test scores (β = 11.79, SE = 2.78, p < .001), lower single-leg stance test (β = 4.20, SE = 1.70, p < .05), and lower 10-m walk test scores (β = -7.13, SE = 2.03, p < .001) than the non-sarcopenia group. 

**Table 3:** Comparisons of physical status for participants by periods and sarcopenia Values expressed as median, first and third quartiles, depending on the duration of Aomori exercises and the presence or absence of sarcopenia. Please refer to the results of the Generalized Linear Mixed Model (GLMM) in Table 4. ^†^p < 0.05 for Mann-Whitney U test, between-group. FTSST; 5 sit-to-stand tests; SPPB; Short Physical Performance Battery. FTSST; Five-times-sit-to-stand test, SPPB; Short Physical Performance Battery, SRT; Sit and Reach Test, SLST; Single Leg Stance Test, AIC; Akaike's Information Criterion, BIC; Bayesian Information Criterion

Measurements	Sarcopenia (+), n=9			Sarcopenia (–), n=15		
	Baseline	One month	Three month	Baseline	One month	Three month
Grip Power (kg)	17.4 (14.3, 21.7)	15.4 (13.4, 19.8)	15.8 (15.6, 25.0)	20.5 (19.1, 22.5)	20.1 (17.3, 23.8)	24.4 (20.9, 26.4)
FTSST (sec)	15.6 (12.9, 19.2)^†^	13.7 (12.7, 15.8)^†^	9.0 (8.9, 10.0)	7.9 (7.3, 10.9)	7.7 (7.5 - 9.1)	7.1 (6.7, 8.2)
Sit-up Test (times)	.0 (.0, .0)	.0 (.0, .0)	.0 (.0 - .0)	.0 (.0, 8.5)	.0 (.0 - 8.5)	.0 (.0, 12.0)
SPPB	8.0 (6.5, 8.0)^†^	9.5 (9.0, 10.0)^†^	11.0 (11.0 - 11.0)	12.0 (11.0, 12.0)	12.0 (11.0 - 12.0)	12.0 (12.0, 12.0)
SRT (cm)	25.5 (22.3, 30.0)	30.0 (20.0, 33.0)	35.5 (32.8 - 44.8)	37.0 (33.0, 40.0)	35.0 (30.0 - 38.0)	37.0 (35.0, 44.0)
SLST (sec)	.7 (.5, .9)^†^	1.8 (1.2, 2.1)	2.2 (1.9 - 2.9)	3.0 (2.1, 5.3)	2.3 (1.9 - 4.5)	3.1 (2.1, 6.3)
10-m Walk Test (sec)	10.0 (9.0, 13.0)^†^	10.3 (9.0, 11.7)^†^	8.6 (8.0 - 10.0)	6.0 (5.5, 6.4)	5.6 (5.2, 6.5)	5.3 (5.1, 5.9)

**Table 4:** Estimated physical status for participants by periods and sarcopenia using GLMM The results from the Generalized Linear Mixed Model (GLMM) for the various performance metrics are summarized. Each metric was analyzed with "periods" as the temporal factor and "sarcopenia" as a fixed factor. Statistics presented by GLMM for duration and by the presence or absence of sarcopenia are expressed as means (standard error). Intercept, Periods, and Sarcopenia are indicated coefficients as β values for main effects. Periods × Sarcopenia is represented as interaction. Please refer to the results for physical status by group and time period shown in Table 3. *p < 0.05 for GLMM. FTSST; 5 sit-to-stand tests; SPPB; Short Physical Performance Battery. FTSST; Five-times-sit-to-stand test, SPPB; Short Physical Performance Battery, SRT; Sit and Reach Test, SLST; Single Leg Stance Test, AIC; Akaike's Information Criterion, BIC; Bayesian Information Criterion, LL; Log-Likelihood

Measurements	Intercept (β)	Periods	Sarcopenia	Interaction	AIC	BIC	LL
Grip Power (kg)	20.97 (1.90)*	1.31 (.77)	3.94 (1.38)*	.53 (.55)	405.43	411.93	-197.71
FTSST (sec)	6.22 (0.93)*	.34 (.46)	-6.95 (.98)*	.84 (.60)	370.73	377.23	-181.36
Sit-up Test (times)	.82 (.85)	.35 (.33)	2.74 (1.06)*	.85 (.39)	376.83	383.33	-184.42
SPPB	11.43 (.70)*	.16 (.29)	3.68 (.97)*	.93 (.43)*	299.79	306.29	-146.89
SRT (cm)	36.65 (1.83)*	.87 (.68)	11.79 (2.78)*	1.39 (.81)	486.00	492.50	-239.99
SLST (sec)	5.15 (2.42)	.52 (1.98)	4.20 (1.70)*	.74 (1.00)	475.91	443.15	-153.55
10-m Walk Test (sec)	6.65 (2.66)	.67 (1.23)	-7.13 (2.03)*	-1.05 (1.20)	392.75	400.84	-162.73

Exploratory outcomes

A Mann-Whitney U test was conducted to compare the differences between the sarcopenia and non-sarcopenia groups across different time periods (baseline, one month, and three months) for physical variables (see Table 3). At baseline, significant differences were found in FTSST (U = 54.5, p = 0.02, r = 0.91), SPPB (U = 5.0, p = 0.02, r = 0.20), single-leg stance test (U = 4.5, p = .01, r = .08), and 10-m walk test (U = 52.0, p = .03, r = .87) scores. After one month, significant differences were noted in FTSST (U = 50.0, p = 0.01, r = 0.96), SPPB (U = 6.0, p = 0.03, r = 0.25), and 10-m walk test (U = 48.0, p = .0147, r = 0.92) scores. No significant differences were observed after three months for any of the variables.

## Discussion

The results of this study suggest that three months of daily short-term exercise can bring older adults with sarcopenia closer to the exercise capacity of older adults without sarcopenia. Sarcopenia is a major factor that contributes to decline in physical ability among the elderly [18]. The improvements observed in the sarcopenia group during the study period indicate that Aomori gymnastics can effectively enhance physical activity in elderly individuals with sarcopenia. Implementing the Aomori gymnastics program in communities and care facilities could provide a practical, low-cost method to improve the physical function and quality of life of the elderly. Healthcare providers and caregivers should consider incorporating regular physical activity habits into care plans to effectively address and manage sarcopenia.

Adherence was higher among those residing in facilities (Group 3) and lower among those living at home and gathering at public facilities (Groups 1 and 2). This discrepancy can be attributed to the climate in Aomori Prefecture, which ranks among the highest in Japan for snowfall, with an annual average of approximately 800 cm. Elderly individuals living at home faced challenges in accessing exercise venues during the winter months (January to March) owing to poor weather conditions. Activity levels were reported to reduce among the elderly in Japan during the snowfall season [19] because icy roads and the risk of falls discourage outdoor activities [20]. Aomori gymnastics can be performed indoors, in groups, and while watching video guides, making it suitable for remote or home-based exercise programs for the elderly. Reports have indicated that online exercise programs can improve compliance [21], although the use of online devices is challenging for the very elderly, necessitating regional initiatives. This approach can mitigate the issues caused by harsh weather conditions. By integrating Aomori gymnastics into video conferences or exercise apps, participants can conveniently engage in guided exercise sessions from their homes [22,23]. Additionally, involving family members as exercise partners or motivators could enhance adherence among home-dwelling older adults. Local community volunteers could also be trained to provide in-home guidance for those unable to use technology independently. Establishing a telephone support system where healthcare providers periodically check in with participants may further promote accountability and address any concerns that arise during the exercise program.

Unlike conventional resistance training programs that primarily target muscle strength for sarcopenia management, Aomori gymnastics offers a comprehensive, culturally-relevant approach that addresses multiple physical domains simultaneously. Traditional interventions for sarcopenia often require specialized equipment, trained personnel, or high-intensity exercises that may limit adherence among older adults. In contrast, our program integrates culturally meaningful elements from the "Nebuta" festival, which potentially enhances motivation and emotional engagement while requiring minimal equipment. The accessibility and enjoyable nature of these exercises promote consistent participation, which is crucial for the elderly population in regions with severe weather conditions like Aomori, where outdoor activity can be limited for extended periods.

Our findings highlight important differences between community-dwelling participants and those in long-term care facilities that extend beyond sarcopenia status. While our primary analysis focused on sarcopenia as the distinguishing factor, the implementation environment likely played a crucial role in observed outcomes. Long-term care facilities provide structured routines, dedicated spaces for exercise, and staff who can offer reminders and encouragement, potentially explaining the higher adherence rates in Group 3, regardless of physical status. Community-dwelling participants face additional barriers, including transportation challenges, competing priorities, and lack of supervision, which were exacerbated by Aomori's severe winter conditions. Future research should specifically design comparative analyses between community and institutional settings to identify optimal implementation strategies for each environment. Such research could investigate how social support networks, environmental factors, and program delivery methods might be tailored to improve adherence among community-dwelling older adults, particularly during challenging weather conditions, while maintaining the benefits observed in institutional settings.

Although this study included only women, this was not a coincidence. In Japan, older men tend to participate less in group activities than older women [24]. This behavioral trait likely influenced the lack of male participants in our study. Additionally, the elderly women in our study showed improvements in physical function through continued exercise. In such group programs, women often show a greater increase in physical activity levels than men [25], which is partly because older men typically have higher baseline physical activity levels [26]. Thus, even when performing the same exercises designed with safety in mind, physical function improvements are particularly noted in women. Exercise programs of varying intensities for men and women should be developed in future studies to encourage male participation in group exercise programs.

This study had several limitations. First, the sample size was relatively small (n=24), particularly the extremely low number in Group 2 (n=2), which may have limited the statistical power of the study to detect significant differences between the groups. While our post-hoc power analysis indicated adequate power for our primary outcomes (post-hoc power critical z=1.64, 1-β=.84, and effect size h=.20 from z-test), caution is warranted when generalizing these results to the broader elderly population with sarcopenia. Future research should prioritize larger-scale, multi-center data collection efforts to validate our findings across diverse elderly populations and geographic regions in Japan. Second, despite comparing participants with sarcopenia or no sarcopenia within the same cohort of exercise program attendees, the possibility of selection bias cannot be eliminated. Participants chose to participate in the program; thus, unmeasured factors, such as motivation level, may have differed between the sarcopenia and non-sarcopenia groups, influencing the results. Third, the three-month observation period only allowed for the assessment of short-term effects. The long-term sustainability of the improvements in physical function remains unclear. A longer follow-up period is warranted to evaluate whether the benefits remain for extended periods. Additionally, we did not investigate participants’ baseline nutritional status or nutritional information. Fourth, as this study was not conducted in a clinical setting, we were unable to collect comprehensive medical parameters. The lack of detailed data on comorbidities, pharmacological treatments, and objective muscle mass measurements through imaging techniques (such as dual-energy X-ray absorptiometry or bioelectrical impedance analysis) limited our ability to accurately diagnose sarcopenia according to the full Asian Working Group for Sarcopenia criteria. Instead, we relied on functional measures (SPPB scores and FTSST), which may have affected diagnostic precision and our ability to control for medical confounding factors. Additionally, we did not investigate participants' baseline nutritional status or nutritional information. Combining exercise with nutritional management has been reported to ameliorate frailty in the elderly within three months [27]. We could not analyze the impact of nutrition on the physical function improvements we observed. Incorporating nutritional interventions along with exercise programs could enhance the achievement of functional gains. Moreover, the effect of the exercise on individuals with cognitive impairment was not examined because individuals with cognitive issues were not included. Previous evidence indicates that aerobic exercise improves both physical and cognitive function in mild cognitive impairment [28,29]. For institutionalized elderly individuals with cognitive deficits, continuous encouragement and support may be needed to promote compliance with the exercise routine and may extend the benefits to the cognitive domains. Further research in this population is warranted.

Another limitation of our study was that all participants with sarcopenia were from the long-term care facility group (Group 3), while none of the community-dwelling participants (Groups 1 and 2) presented with sarcopenia. This distribution limits our understanding of how Aomori gymnastics might specifically benefit community-dwelling older adults with sarcopenia, who represent an important target population for preventive interventions. Future studies should actively recruit community-dwelling elderly with sarcopenia to better evaluate the program's effectiveness in this population and to determine whether similar improvements in physical function can be achieved. This would provide valuable insights for developing targeted community-based exercise programs that could potentially delay institutionalization and maintain longer independence among vulnerable older adults living at home.

## Conclusions

This study suggests that Aomori gymnastics can improve physical function in frail older women. Further research is needed to develop strategies for implementing Aomori gymnastics, regardless of the season of the year and participant residence, to effectively prevent care dependence among the elderly.
